# Refractory diarrhea in a patient with Sjogren’s syndrome: A case report

**DOI:** 10.3389/fnut.2023.1086967

**Published:** 2023-02-07

**Authors:** Liling Xu, Ming Gui, Chuanzheng Sun, Vicky Yau, Chenyu Sun, Jing Qi

**Affiliations:** ^1^Department of Emergency, The Third Xiangya Hospital of Central South University, Changsha, Hunan, China; ^2^Department of Nephrology, The Third Xiangya Hospital of Central South University, Changsha, Hunan, China; ^3^Division of Oral and Maxillofacial Surgery, NewYork-Presbyterian/Columbia University Irving Medical Center, New York, NY, United States; ^4^Department of Thyroid and Breast Surgery, The Second Hospital of Anhui Medical University, Hefei, China; ^5^AMITA Health Saint Joseph Hospital Chicago, Chicago, IL, United States

**Keywords:** diarrhea, Sjogren’s syndrome, cachexia, refeeding syndrome, nutrition

## Abstract

We present the case of a 66-year-old man with no abdominal symptoms other than chronic refractory diarrhea. Other causes for diarrhea were excluded. The positive results of anti-SSA antibodies, Schirmer’s test, and the biopsy of minor salivary glands confirmed the diagnosis of Sjogren’s syndrome. Moreover, during the course of treatment, the patient developed refeeding syndrome. His diarrhea and nutrition resolved with initiation of glucocorticoids. This case highlights that chronic refractory diarrhea can be the chief complaint and most severe symptom in patients with Sjogren’s syndrome.

## Introduction

Sjogren’s syndrome is an autoimmune disease characterized by hypofunction of salivary and lacrimal glands and possible multiorgan manifestations (1). Occasionally, extraglandular manifestations in Sjogren’s syndrome may precede dry symptoms, leading to misdiagnosis. Sjogren’s syndrome without other connective tissue diseases is called pSS, with a female-to-male predominance of 9:1 and peak incidence at approximately 50 years of age (2). It is relatively rare in old men. Chronic diarrhea has been described in up to 9% of patients with Sjogren’s syndrome (3), but it is rare that chronic refractory diarrhea can be the chief complaint and most severe symptom. The patient received enteral nutrition therapy after admission, which cause refeeding syndrome (RFS). RFS is a potentially fatal complication that occurs when a malnourished organism is reintroduced to substances (4). The body will undergo a sharp change from fat metabolism to carbohydrate metabolism in the process, causing increased insulin secretion, resulting in the transfer of magnesium, potassium, and phosphorus ions into cells. As a result, low levels of serum electrolytes will occur. Patients should receive parenteral electrolyte replacement and additional vitamin B1 supplements during which serum electrolyte levels need to be closely monitored.

## Case report

A 66-year-old man with a 4-year history of refractory diarrhea was admitted to the Department of Gastroenterology, The Third Xiangya Hospital of Central South University, with complaints of yellow-appearing diarrhea occurring 4–6 times per day. He also reported 10-kg of weight loss over the last 8 months. He denied any abdominal pain, abdominal distention, nausea, vomiting, fever, hematochezia, or melena. The patient was initially misdiagnosed with anorexia nervosa due to poor appetite and low BMI, but later revealed his reluctance to eat was secondary to his fear of developing diarrhea after food ingestion. Due to his malnourished state, a nasogastric tube was inserted under EGD guidance for enteral nutrition.

The patient was diagnosed with non-atrophic gastritis and colonic polyps a year ago and subsequently had the colonic polyps removed by high-frequency electrocoagulation 6 months later. He denied any history of medications, including anti-hypertensives, magnesium supplements, and non-steroidal anti-inflammatory drugs (NSAIDS). There was no known history of drug or food allergies. The patient had a 25-year history of smoking tobacco but had quit 3 months prior to admission. He only occasionally drank alcohol and denied any exposure to radioactive or toxic materials.

On admission, the patient’s temperature as 36.5°C, with heart rate of 65 beats per minute, and respiratory rate of 20 breaths per minute. His blood pressure was 99/67 mmHg, and oxygen saturation was 98% on room air. The patient was 41 kg, 167 cm, hence BMI of 14.7. On physical exam, cardiac and respiratory examinations were unremarkable. The abdomen was soft, non-tender on palpation, and without rebound tenderness, or masses. The liver and spleen were impalpable.

The patient’s white-cell count (WBC) was 3,660 per cubic millimeter (29.7% neutrophils, 54% lymphocytes, 8.5% monocytes), hemoglobin (Hgb) was 9.8 g per deciliter (g/dl), and the platelet count was 159,000 per cubic millimeter. The alanine aminotransferase (ALT), aspartate aminotransferase (AST), total bilirubin (T. Bili), blood urea nitrogen (BUN), creatinine (Cr), serum glucose level, lipid panel, thyroid stimulating hormone (TSH), C-reactive protein (CRP), and electrolyte levels were all within normal limits. Serum CEA, AFP, CA12-5, and CA19-9 were also normal. Albumin level was slightly decreased to 3.4 g per deciliter (g/dL). Fecal elastase-1 level was 165.77 μg/g. Levels of serum gastrin 17 and fecal calprotectin were normal (Table 1). The enzyme linked immunosorbent assay (ELISA) test for IgG antibodies to 90 unique food allergens (Allerquant™ 90G ELISA Kit, BIOMERICA) in his serum was negative.

**TABLE 1:** Laboratory data.

Variable	Reference range, adults	On admission
**Blood**		
Hemoglobin (g/dl)	13.0–17.5	9.8
Platelet count (per μl)	125,000–350,000	159,000
White-cell count (per μl)	3500–9500	3,660
**Differential count (%)**		
Neutrophils	40.0–75.0	29.7
Lymphocytes	20.0–40.0	54.0
Monocytes	3.0–10.0	8.5
Alanine aminotransferase (U/L)	9.0–50.0	13.0
Aspartate aminotransferase (U/L)	15.0–40.0	34.0
Total bilirubin (μmol/L)	3.4–20.5	12.4
Albumin (g/dl)	4.0–5.5	3.4
Urea nitrogen (mmol/L)	3.1–9.5	4.88
Creatinine (μmol/L)	57.0–111.0	59.0
Glucose (mmol/L)	3.9–6.1	4.23
Thyroid stimulating hormone (μIU/ml)	0.27–4.2	2.97
C-reactive protein (mg/dl)	0–0.6	<0.5
**Lipid (mmol/L)**		
Total cholesterol	<4.14	2.17
Triglyceride	<1.7	0.55
**Electrolytes (mmol/L)**		
Sodium	137.0–145.0	135.7
Potassium	3.5–5.3	3.43
Chloride	99.0–110.0	101.9
Calcium	2.2–2.7	2.22
**Tumor markers**		
CEA (ng/ml)	0–5	4.18
AFP (ng/ml)	0–20	2.39
CA12-5 (U/ml)	0–35	13.34
CA19-9 (U/ml)	0–34	8.49
Gastrin 17	≤5.7	0.47
**Feces**		
Elastase-1 (pmol/l)	>200	165.77
Calprotectin (ug/g)	<200	<30

Meanwhile, we reviewed his test results from an outside hospital. The esophagogastroduodenoscopy (EGD) and colonoscopy a year ago showed non-atrophic gastritis and colonic polyps. Fluorine 18 fluorodeoxyglucose (FDG) PET/CT showed no abnormal FDG uptake in gastrointestinal tract, pancreas, liver, spleen, gallbladder, kidneys, adrenal glands, or bladder.

The laboratory data were consistent with mild-moderate pancreatic exocrine insufficiency, given the elastase-1 level was less than 200 μg/g. The patient was treated by oryz-aspergillus enzyme and pancreatin tablet (Combizym, Daiichi-Sankyo Europe, Munich, Germany), however, his diarrhea did not improve. On the fifth day after admission, the patient spiked a fever (Tmax 39.5°C) and reported chills. He denied cough, sputum, canker sores, pollakiuria, and dysuria. Faint bibasilar rales were noted on auscultation. The white-cell count was 6,530 per cubic millimeter (56.1% neutrophils, 34.6% lymphocytes, 6.3% monocytes) and CRP 0.99 mg/dL (normal value, < 0.5). The erythrocyte sedimentation rate (ESR) was 33 mm per hour (normal range, 0–28), the procalcitonin (PCT) level was 2.25 ng per deciliter (ng/dL), and blood cultures were obtained. The patient received empiric antibiotic treatment with piperacillin-tazobactam for possible pneumonia.

Two days later, the fever resolved. However, on the twelfth day, the patient had a fever again, with peak temperatures of 40.0°C. The white-cell count was 2,070 per cubic millimeter (59.6% neutrophils, 30.6% lymphocytes, 5.1% monocytes), the hemoglobin level 8.6 g/dL, and the platelet count 149,000 per cubic millimeter. Liver and renal function measurements remained normal except for a lower albumin level of 3.2 g/dL. Comprehensive metabolic panel (CMP) showed potassium of 4.31 mmol per liter (mmol/l), sodium of 111.4 mmol/l (normal range, 137–147), chloride of 79 mmol/l (normal range, 99–109), phosphorus of 0.46 mmol/l (normal range, 0.85–1.51), calcium 1.81 of mmol/l (normal range, 2.2–2.7), and magnesium of 0.63 mmol/l (normal range, 0.75–1.02). The anion gap was 9.7 mmol/l. The PCT level was 2.87 ng dL and CRP was 2.66 mg/dL (normal range, < 0.5). Urinalysis showed no protein, red cells, or white cells per high-power field. Fecal studies showed no fecal leukocytes, ova or parasites, and the fecal occult blood test (FOBT) were negative. A sputum culture, two sets of blood and stool cultures were negative, as were serologic tests for mycoplasma, chlamydia, varicella–zoster virus, adenovirus, coxsackie virus, influenza virus, epidemic hemorrhagic fever virus, mumps virus, respiratory syncytial virus, measles virus and echovirus. Testing for HIV and HCV antibody, HBsAg and clostridium difficile (C. diff) toxin were negative. The T-cell spot test for tuberculosis infection (T-SPOT.TB), and polymerase-chain-reaction (PCR) assays for epstein-barr virus and cytomegalovirus were all negative. 1,3-β-D-glucan and galactomannan content detection were within normal range. High resolution computed tomography (HRCT) of the chest showed bilateral moderate pleural effusion and pneumonia. CT of abdomen with of contrast showed an enlarged liver without masses and a small amount of ascites.

The antimicrobial regimen was replaced with intravenous meropenem. Meanwhile, the patient began to receive 50% saline orally, 500 ml of hypertonic saline (4.5%) intravenously, and 10 ml of 2.16 g sodium glycerophosphate in 500 ml dextrose 5% in water intravenously. The level of blood sodium rose by 9 mmol/l on the first day, then by 3–5 mmol/l daily until it was normalized. The hypophosphatemia was also gradually resolved.

**FIGURE 1:**
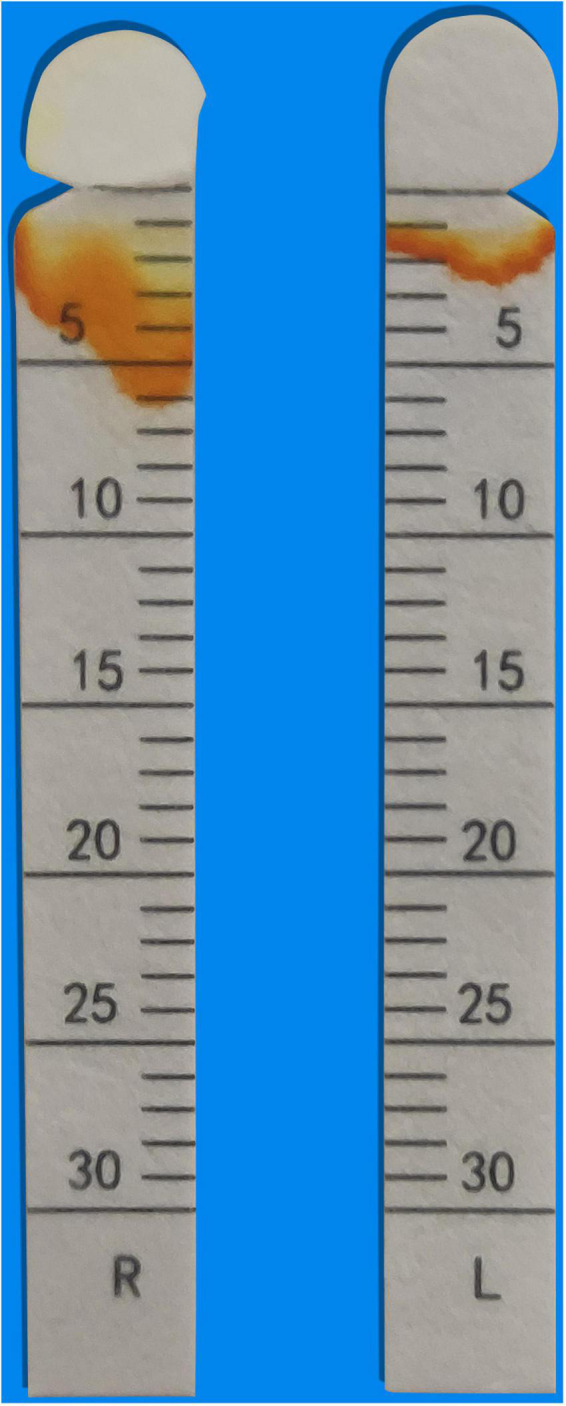
Schirmer’s test. Schirmer test of < 5 mm per 5 min (left eye).

**FIGURE 2:**
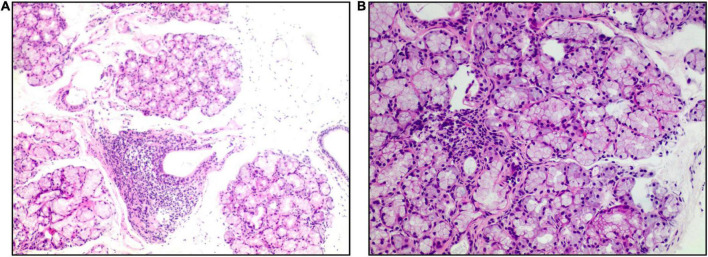
Salivary gland biopsy (hematoxylin and eosin). Panel **(A)** (×40 magnification) shows multiple labial glandular lobules, local atrophy of individual acini and interstitial fibrosis. Panel **(B)** (×100 magnification) shows a lymphocytic focus.

The fact of simultaneous pleural effusion, peritoneal effusion, and hepatomegaly raised the possibility of the diffuse serosal inflammation (e.g., SLE). So some tests for connective tissue diseases were done. The C3 level was 0.6 g per liter (g/L) (normal range, > 0.79), and the C4 level was 0.2 g/L (normal range, > 0.16). Test for ANA (nuclear granular type) was present at a titer of 1:320. Test for anti-SSA antibodies and anti-mitochondrial antibodies were positive. Anti-SSB antibodies, antineutrophil cytoplasmic antibodies (ANCA), rheumatoid factor, cyclic citrullinated peptide (CCP) antibodies and anti-dsDNA antibodies were not detected. The level of IgG4 was normal. After focused history taken for rheumatic and autoimmune diseases, a 9-year history of a persistent dry mouth was reported by the patient, so Schirmer’s test was positive (Figure 1). Then biopsy of minor salivary glands was performed, showing focal lymphocytic sialadenitis (FLS). Focus score was 1 (Figure 2).

A presumptive diagnosis of primary Sjogren’s Syndrome related fever and diarrhea was made. The patient’s diarrhea resolved and fever subsided with initiation of glucocorticoids (methylprednisolone tablets orally, 12 mg/day). By the time he was discharged from our hospital, his bowels had been regular and the level of electrolytes returned to normal range. He felt well and gained 5.5 kg in weight at 1-month follow-up.

## Discussion

Chronic diarrhea represents a complex diagnostic challenge. The manifestations that prompted consideration of rheumatic diseases in this case report were the fact that our male patient developed fever, leukopenia, and CT imaging found evidence of serositis and hepatomegaly. The positive results of anti-SSA antibodies, Schirmer’s test, and the biopsy of minor salivary glands confirmed the diagnosis of Sjogren’s Syndrome.

There are a variety of gastrointestinal (GI) manifestations that have been described in Sjogren’s syndrome. These may occur due to lymphocytic infiltration of the GI mucosa or exocrine glands, autonomic neuropathies or the development of associated autoimmune diseases that occur with increased frequency in Sjogren’s syndrome (5). In fact, it’s sometimes difficult to tell whether diarrhea is caused by the Sjogren’s syndrome itself or is associated with other diseases such as microscopic colitis (MC). In this patient, refractory diarrhea is the chief complaint and most severe symptom. Tumors and inflammatory bowel diseases (IBD) were ruled out by reviewing his previous EGD reports and images. Viral hepatitis was excluded, because the negative HCV antibody and HBsAg. However, no further colonoscopy, double-balloon enteroscopy (DBE), or biopsy of GI tract were performed, because the patient was too weak to complete the bowel preparation. Therefore, it is not clear what type of chronic colitis is associated with Sjogren’s syndrome in the present case.

Most pancreatic involvement in Sjogren’s syndrome relates to pancreatic exocrine insufficiency and is typically asymptomatic (6). Hepatomegaly occurs in 11–21% of patients with Sjogren’s syndrome and may be caused by primary biliary cholangitis (PBC), autoimmune hepatitis (AIH), and sclerosing cholangitis (SC) (2, 7). Although these findings appeared in this patient, the absence of characteristic dry mouth and eye made it hard to immediately establish the diagnosis of Sjogren’s Syndrome. The possibility of connective tissue diseases was considered in view of the patient’s unsatisfactory response to pancreatin supplementation, subsequent recurrent fever, and pleural and abdominal effusion. IgG4-related diseases were excluded, because the normal range of IgG4. The diagnosis of Sjogren’s syndrome was ultimately confirmed by the positive Schirmer’s test, serology, and the biopsy of minor salivary glands.

The patient developed severe hyponatremia, hypochloremia, and hypophosphatemia after receiving artificial enteral refeeding. The refeeding syndrome was considered on basis of his malnutrition and anorexia. Refeeding syndrome is defined as the potentially fatal shifts in fluids and electrolytes that may occur in malnourished patients receiving artificial refeeding (whether enterally or parenterally). The hallmark biochemical feature of refeeding syndrome is hypophosphatemia (4). It is often not recognized or inappropriately treated, especially on general ward (8). We found no case reports of Sjogren’s syndrome with refeeding syndrome. Hypophosphatemia in hospitalized patients is ideally treated with intravenous supplementation. The patient’s level of electrolytes returned to normal through oral and peripheral venous supplement of sodium, phosphorus, and multivitamins. It must be noticed that the rate of sodium correction should not exceed 8–10 mmol/L in 24 h to avoid acute increase in extracellular tonicity which can cause osmotic demyelination syndrome (ODS) (9). He did not develop ODS during correction of electrolyte imbalance with the proper rate of sodium replacement.

Sjogren’s syndrome is difficult to diagnose and manage. Recent evidence reveals Sjogren’s syndrome as a true orphan disease, without any efficacious agent. In daily practice, therapeutic decisions are often based on personal experience and expert recommendations. EULAR recommends that systemic therapies (glucocorticoids, immunosuppressive agents, antimalarials, intravenous immunoglobulins, biologics) may be considered for patients presenting with a moderate disease activity score (score > 5) (10, 11). In this case, his EULAR Sjogren’s Syndrome Disease Activity Index (ESSDAI) was 7, so glucocorticoids were considered as the first- line option, and was given with a satisfactory outcome.

## Conclusion

This case highlights the importance of considering the possibility of Sjogren’s syndrome in persons presenting with chronic refractory diarrhea and alerting malnourished patients to the refeeding syndrome after starting artificial feeding. Earlier diagnosis and treatment can prevent patients from cachexia and result in a favorable clinical outcome.

## Data availability statement

The original contributions presented in this study are included in this article/supplementary material, further inquiries can be directed to the corresponding authors.

## Ethics statement

Written informed consent was obtained from the individual(s) for the publication of any potentially identifiable images or data included in this article.

## Author contributions

LX, CyS, and JQ researched the data and wrote the manuscript. MG and CzS researched the data and contributed to the discussion. VY reviewed the manuscript. All authors have read and approved the manuscript.
